# Prevalence and Determinants of Gastroesophageal Reflux Disease and the Risk Factors Among Adult Patients Attending Al-Iskan Primary Health Care Center in Makkah, 2020

**DOI:** 10.7759/cureus.10535

**Published:** 2020-09-18

**Authors:** Hamza Halawani, Shadi Banoon

**Affiliations:** 1 Family Medicine, Aliskan Primary Health Care, Makkah, SAU; 2 Family Medicine, Primary Health Care, Ministry of Health, Makkah, SAU

**Keywords:** gerd, makkah, prevalence, risk factors, primary health care, gastroesophageal reflux disease

## Abstract

Globally, gastroesophageal reflux disease (GERD) has a potentially high prevalence, with a wide rate of variability across different populations due to inconsistency in the risk factors. Hence, a cross-sectional study was conducted using a self-administered, structured questionnaire at Al-Iskan Primary Health Care (PHC) Center to investigate the prevalence rate and associated factors in Makkah Almukarramah, the west of Saudi Arabia. The study included 339 participants. The mean age for participants was 39.5 ± 15.5 years, and the range was from 18 to 84 years. Male participants were 247 (72.9%) and females were 92 (27.1%). Participants were diagnosed with GERD on achieving a GERD questionnaire score of ≥8. In our sample, 59 (17.4%) were diagnosed with asymptomatic GERD. The represented logistic regression shows that family history of GERD, marital status (divorce), smoking, physical activity frequency, tomatoes/tomato-based foods, salty foods, acidic drinks, frequent use of analgesics, and nonsteroidal anti-inflammatory drugs (NSAIDs) consumption shows statistical significance and association (P < 0.05) with increased risk of symptomatic GERD. Conclusively, the results show that GERD is prevalent in Makkah with the presence of modifiable risk factors which can significantly affect the ascendency of the disease.

## Introduction

Gastroesophageal reflux disease (GERD) is reported as a common disorder of the upper part of the gastrointestinal tract. It is one of the functional gastrointestinal disorders that implies the chronicity and recurrence of the clinical picture [1]. If left untreated, GERD may lead to a severe prognosis with serious morbidities, complications, and economic burdens that requires lifestyle modifications, surgical intervention, and long-term management regimens [2]. Asymptomatic gastric refluxes is usual in normal population, however, frequent occurrence of this phenomenon associated with specific symptomatology causes the disorder [3].

The pathophysiology of the disease is multifactorial, and involves the gastroesophageal junction [4]. These factors mainly include the increased pressure and compliance on the junction, leading to pathological regurgitation of the gastric acidic content, which mainly induces the pathology and clinical picture of the disease [5]. Additionally, a variety of risk factors are reported for individuals who developed the disorder. These include genetic factors that determine the amount of acid release, body mass index (BMI), *Helicobacter pylori* infection, and many others, including individual lifestyles such as diet components, personal habits, and medication intake history [6].

Globally GERD has a potentially high prevalence with a wide rate variability across different populations due to the inconsistency in the risk factors. The results of a recently published meta-analysis of 96 studies from 37 countries estimated the global prevalence rate of GERD as 13.98%, with the highest rates in North America (19.55%) and Europe (14.12%), while in Asia, the estimated rate was 12.92% in 54 studies [7]. Moreover, the prevalence rate was tremendously variable among the different countries in Asia. Iran had the highest incidence rate (18.43%) while in Japan and China the rate decreased to 13.81% and 4.16% as reported by 16, 10, and seven studies, respectively. However, the same meta-analysis could not estimate the prevalence rate in Saudi Arabia due to the limited number of investigations that reported the disorder. As far as we know, a few published investigations have estimated the ascendence of GERD in Saudi populations. In the southwestern region, Kariri et al. [8] reported a prevalence rate of 32.2% based on GERD questionnaire (GerdQ) scores, and in 2016, Alsuwat et al. [9] reported a rate of 28.7% in the general Saudi population. Therefore, due to a few studies that reported the prevalence rate of GERD in Saudi Arabia and the probability of possible variations as reported in other countries, we aim to investigate the frequency rate and associated factors in Makkah Almukarramah in the west of Saudi Arabia. 

## Materials and methods

Study design and population

A cross-sectional study was conducted using a self-administered, structured questionnaire at Al-Iskan Primary Health Care (PHC) Center, a training center for the family medicine program in the Makkah Al-Mukarramah region. For this study, we recruited patients who attended the Al-Iskan PHC center in Makkah during July 2020. The involvement criteria were adult patients (>18 years old) with no restrictions in terms of gender and nationality. Pediatric patients, pregnant women, adults who had not completed the questionnaire, or were found unwilling to participate were excluded. The sample size was calculated using the software by Raosoft, Inc. (http://www.raosoft.com/samplesize.htm). After the calculation, we found that the minimum sample size achieved a precision of ±5%, and a 95% confidence interval (CI) was 337 (after accounting for 10% unresponsive and incomplete data).

Data collection and sampling technique

The sample size was fulfilled by a random sampling technique with each patient having an equal chance, and the researcher chose from the random sample using a random number table. The data was collected utilizing a structured questionnaire developed by the researcher. This was a self-administrated questionnaire completed by the patients in the clinic under direct supervision of the clinicians. Furthermore, it was filled by the clinician in case of illiteracy or difficulty doing it themselves. The first part consisted of questions about the sociodemographic data of the patients, the second part was related to different risk factors and personal habits, and the third part was the gastroesophageal reflux disease questionnaire (GerdQ); a diagnostic tool for GERD [10,11].

GerdQ was composed of six questions, four questions about the positive GERD predictors (heartburn, regurgitation, sleep disturbance due to heartburn and regurgitation, and the use of over the counter [OTC] medications), and two questions about the negative GERD predictors (nausea and epigastric pain). The questionnaire was translated from English to Arabic and was translated by two translators back to English to support the validity of the questionnaire. The scoring of GerdQ depended on the frequency of these symptoms during the last week (less than once, once, two to three times, and four to seven times, respectively), where the scores ranged from 0 to 3 for the positive GERD predictors, and reversed the order for the negative GERD predictors (3 = None). After summation of the scores, the patients who scored an 8 or more were considered positive for GERD. GerdQ sensitivity and specificity for GERD diagnosis was 65% and 71%, respectively [10,11].

Statistical analysis

All data was analyzed using R software, version 4.0.2, using the packages (Rcmdr) and (glm2). All nominal variables were represented as frequencies, and percentages with Chi2 test (or Fisher’s exact test, as appropriate) were used for testing the difference as per the presence or absence of GERD. We used a t-test or Mann-Whitney test based on the distribution of the data (normally distributed or not). Furthermore, we used univariate logistic regression to identify any possible association between GERD incidence and different possible risk factors [12]. Regression results were expressed as odds ratios (ORs) with 95% confidence interval (95% CI).

## Results

Sociodemographic characteristics

Out of 385 questionnaires distributed, 339 were completed generating a response rate of 88%. The sociodemographic characteristics for the valid participants are presented in Table 1. The mean age for participants was 39.5 ± 15.5 years, and ranged from 18 to 84 years. Male participants were 247 (72.9%) and females were 92 (27.1%). Nearly all patients resided in urban areas (97.3%), 41% had a university or higher degree, and 40.4% were employees. As for BMI, which was calculated by the research team, 21.8% of the participants had a normal weight (18.5-24.9 kg/m2), 46.9% were overweight (25-29.9 kg/m2), and 31.3% suffered from obesity (30 kg/m2 or greater).

**Table 1:** Baseline characteristics of the participants GERD: gastroesophageal reflux; SD: standard deviation; *Statistically significant

Variables		GERD		No GERD		Total		P-value
		n= 59 (17.4%)		n= 280 (82.6%)		N= 339 (100%)		
		Count	%	Count	%	Count	%	
Age; Mean ± SD		39.6 ± 12.9		39.5 ± 16.0		39.5 ± 15.5		0.926
Marital Status	Divorced	14	23.7	31	11.1	45	13.3	0.015*
	Married	36	61.0	175	62.5	211	62.2	
	Single	9	15.3	74	26.4	83	24.5	
Gender	Female	18	30.5	74	26.4	92	27.1	0.522
	Male	41	69.5	206	73.6	247	72.9	
Habitat	Rural	2	3.4	7	2.5	9	2.7	0.659
	Urban	57	96.6	273	97.5	330	97.3	
Educational Level	Illiterate	8	13.6	20	7.1	28	8.3	0.082
	Primary/Middle School	5	8.5	57	20.4	62	18.3	
	Secondary School	22	37.3	88	31.4	110	32.4	
	University degree and above	24	40.7	115	41.1	139	41.0	
Occupation	Employee	20	33.9	117	41.8	137	40.4	0.776
	House Wife	7	11.9	33	11.8	40	11.8	
	Retired	8	13.6	26	9.3	34	10.0	
	Student	5	8.5	29	10.4	34	10.0	
	Unemployed	12	20.3	51	18.2	63	18.6	
	Labourer	7	11.9	24	8.6	31	9.1	
Blood Group	A-	3	5.1	8	2.9	11	3.2	0.283
	AB-	2	3.4	11	3.9	13	3.8	
	B-	8	13.6	17	6.1	25	7.4	
	O-	2	3.4	10	3.6	12	3.5	
	A+	1	1.7	11	3.9	12	3.5	
	AB+	3	5.1	12	4.3	15	4.4	
	B+	3	5.1	8	2.9	11	3.2	
	O+	5	8.5	13	4.6	18	5.3	
	I don't Know	32	54.2	190	67.9	222	65.5	
BMI Groups	Normal weight	16	27.1	58	20.7	74	21.8	0.119
	Overweight	31	52.5	128	45.7	159	46.9	
	Obesity	12	20.3	94	33.6	106	31.3	

Gastroesophageal reflux disease prevalence

Participants were diagnosed with GERD if they had a GerdQ score of ≥8. Out of the 339 participants, 59 (17.4%) had symptomatic GERD. While those having a GerdQ score of less than eight were 280 (82.6%) individuals. There are no significant differences in the sociodemographic of GERD patients compared to their healthy peers, except marital status (P = 0.015) (Table 1).

Risk factors for GERD symptoms

The family history of GERD was present in 27 (45.8%) of the GERD patients, and 40 (14.3%) of the participants with no GERD symptoms. About 65% of the participants did not perform 30-minute physical activity at least once/week, and only 21.8% did so one to three times/week. Moreover, nearly half of the participants were smokers (45.7%), consumed fast food (57.8%), and about one-third (35.7%) of them were frequent analgesics users. As shown in Table 2, there are significant differences in all habits and risk factors of GERD and non-GERD participants.

**Table 2:** Comparison of different risk factors between GERD and No GERD participants GERD: gastroesophageal reflux; *Statistically significant < 0.05; **Statistically significant < 0.001

Variables		Final Diagnosis						P-value
		GERD		No GERD		Total		
		Count	%	Count	%	Count	%	
Family History of GERD	No	32	54.2	240	85.7	272	80.2	< 0.001**
	Yes	27	45.8	40	14.3	67	19.8	
Frequency of physical activity; 30min/Week	> 3 times	1	1.7	8	2.9	9	2.7	0.006*
	1-3 times	23	39.0	51	18.2	74	21.8	
	None	30	50.8	190	67.9	220	64.9	
	Once	5	8.5	31	11.1	36	10.6	
Whenever analgesics needed, Which type do you use?	Don't use analgesics	10	16.9	87	31.1	97	28.6	0.001*
	NSAIDs	23	39.0	59	21.1	82	24.2	
	Paracetamol	24	40.7	134	47.9	158	46.6	
	Other analgesics	2	3.4	0	0.0	2	0.6	
How many meals do you eat daily?	< 3 Meals	11	18.6	56	20.0	67	19.8	0.001*
	> 3 Meals	2	3.4	66	23.6	68	20.1	
	3 Meals	46	78.0	158	56.4	204	60.2	
Which food types do you consume frequently?	Chocolate	12	20.3	92	32.9	104	30.7	< 0.001**
	Fatty Foods	23	39.0	129	46.1	152	44.8	
	Spicy Foods	14	23.7	57	20.4	71	20.9	
	Tomatoes/Tomato-based food	10	16.9	2	0.7	12	3.5	
Which drinks do you consume frequently?	Citrus drinks	11	18.6	34	12.1	45	13.3	0.010*
	Coffee	18	30.5	83	29.6	101	29.8	
	Mint	4	6.8	12	4.3	16	4.7	
	Soft drinks	8	13.6	100	35.7	108	31.9	
	Tea	18	30.5	51	18.2	69	20.4	
Is there any relief in GERD/acidity symptoms on using any of the following medications (Omeprazole-Esomeprazole-Lansoprazole-Pantoprazole-Rabeprazole)?	Don't Know	0	0.0	2	0.7	2	0.6	< 0.001**
	Don't use antacids	28	47.5	269	96.1	297	87.6	
	No	6	10.2	2	0.7	8	2.4	
	Yes	25	42.4	7	2.5	32	9.4	
Smoking	No	22	37.3	162	57.9	184	54.3	0.004*
	Yes	37	62.7	118	42.1	155	45.7	
Using salt and/or pickles in your daily meals	No	13	22.0	126	45.0	139	41.0	0.001*
	Yes	46	78.0	154	55.0	200	59.0	
Frequently eating fast food	No	23	39.0	120	42.9	143	42.2	0.584
	Yes	36	61.0	160	57.1	196	57.8	
Frequently using analgesics	No	21	35.6	197	70.4	218	64.3	< 0.001**
	Yes	38	64.4	83	29.6	121	35.7	
Eating high-fiber foods	No	7	11.9	49	17.5	56	16.5	0.289
	Yes	52	88.1	231	82.5	283	83.5	

Logistic regression represented in Table 3 shows that family history of GERD, marital status (divorce), smoking, physical activity frequency, tomatoes/tomato-based foods, salty foods, acidic drinks, frequent analgesics usage, and nonsteroidal anti-inflammatory drugs (NSAIDs) consumption shows statistical significance and association (P < 0.05) with increased risk of symptomatic GERD. On the other hand, having frequent meals (>3) shows a statistically significant reduction in the risk of symptomatic GERD. In contrast, age, gender, BMI group, fast foods (not shown), and high-fiber food consumption (not shown), did not show statistical significance concerning GERD (P > 0.05).

**Table 3:** Logistic regression of the most important risk factors GERD: gastroesophageal reflux; BMI: body mass index; SE: standard error; *Statistically significant < 0.05; **Statistically significant < 0.001

Predictor	Estimate	SE	Z	P-value	Odds ratio	Lower	Upper
Family History of GERD							
Yes – No	1.620	0.31	5.2	5.06	2.75	9.33
Age	0.001	0.01	0.08	0.935	1	0.98	1.02
Marital Status							
Divorced - Single	1.310	0.48	2.75	0.006*	3.71	1.46	9.47
Married - Single	0.530	0.4	1.32	0.186	1.69	0.78	3.69
Gender							
Male - Female	-0.200	0.31	-0.64	0.522	0.82	0.44	1.51
BMI Groups							
Overweight - Normal Weight	-0.130	0.35	-0.38	0.707	0.88	0.45	1.73
Obese - Normal Weight	-0.770	0.42	-1.85	0.064	0.46	0.2	1.05
Smoking							
Yes - No	0.840	0.3	2.83	0.005*	2.31	1.29	4.12
Frequency of physical activity; 30 min/Week							
1-3 times – None	1.050	0.32	3.29	2.86	1.53	5.34
> 3 times – None	-0.230	1.08	-0.22	0.829	0.79	0.1	6.56
Once – None	0.020	0.52	0.04	0.967	1.02	0.37	2.83
How many meals do you eat daily							
< 3 Meals – 3 Meals	-0.390	0.37	-1.06	0.287	0.67	0.33	1.39
> 3 Meals – 3 Meals	-2.260	0.74	-3.07	0.002*	0.10	0.02	0.44
Which food types do you consume frequently							
Chocolate – Fatty Foods	-0.310	0.38	-0.82	0.412	0.73	0.35	1.54
Spicy Foods – Fatty Foods	0.320	0.37	0.86	0.392	1.38	0.66	2.87
Tomatoes/Tomato-based Foods – Fatty Foods	3.330	0.81	4.13	28.04	5.77	136.38
Using salt and/or pickles in your daily meals							
Yes – No	1.060	0.34	3.16	0.002*	2.90	1.5	5.6
Which drinks do you consume frequently							
Citrus drinks – Soft drinks	1.400	0.51	2.77	0.006*	4.04	1.5	10.89
Coffee – Soft drinks	1.000	0.45	2.22	0.027*	2.71	1.12	6.55
Mint – Soft drinks	1.430	0.68	2.09	0.037*	4.17	1.09	15.93
Tea – Soft drinks	1.480	0.46	3.24	0.001*	4.41	1.8	10.84
Frequently using analgesics							
Yes – No	1.460	0.3	4.83	4.29	2.38	7.76
Whenever analgesics needed Which type do you use							
NSAIDs – Don't use analgesics	1.220	0.41	2.95	0.003*	3.39	1.5	7.64
Paracetamol – Don't use analgesics	0.440	0.4	1.11	0.268	1.56	0.71	3.42

## Discussion

In this cross-sectional study, we aimed to determine the ascendance of GERD among Makkah residents and the associated factors that increased the risk of developing the disorder. According to the analysis of the GerdQ questionnaire results, the GERD frequency rate was 17.4% among the participants. Our results were lower than those reported by Alrashed et al. [13], Alsuwat et al. [9], and Kariri et al. [8] who reported a higher prevalence rate of 23.8%, 28.7%, and 32.2%, respectively, in their Saudi populations. Moreover, much higher incidence rates were also recorded as Alsulobi et al. recorded a rate of up to 61.8%. In the same context, Altwigry et al. [14] confined their population to only Saudi teachers and reported a prevalence rate of 55%. The difference between the reported rates was due to many factors, including the difference in the tools used for patients’ assessments and the individual and environmental associated factors for each patient. Although our study used the GerdQ assessment approach, our reported prevalence rate was more similar to the one reported by Al-Humayed et al. [15] who reported an ascendance rate of 15% in the southern region of Saudi Arabia in an endoscopy-based assessment.

Moreover, to completely understand the difference in prevalence rates, we assessed the associated risk factors with GERD. At first, we did not notice any statistical significance in terms of age. However, Kariri et al. [7] was the only Saudi study to find significance, and supported their finding with another two non-Saudi studies [16,17]. Our results were similar to the results of Alrashed et al. [13], Alsuwat et al. [9], and Alsulobi et al. [18] who also found no significance. Furthermore, we found significance between the GERD and non-GERD groups in terms of marital status (P = 0.015), mainly between the divorced and single patients (P = 0.006), which indicated the fact that emotional disturbances can be a risk factor that may subsequently lead to developing the disorder [19]. These results were consistent with the results of previously published investigations [8,9]. Even though the presented percentage of males and females in our study was not representative of the population, it followed a similarity when compared to major studies with no significance found in terms of gender [8,9,18]. However, Alrashed et al. [11] indicated that the male population was at higher risk for developing GERD and supported their results through a report in India that revealed similar findings [13]. 

We also developed a univariate logistic regression model for better assessment of the most important risk factors. Our analysis showed that having a family history of GERD was a significant risk factor for developing the condition at a later stage (P < 0.001). Our results were similar to the study by Alkhathami et al. [20] which reported that positive family history was present in 39.3% of patients diagnosed with GERD. In the same context, various previous investigations have revealed that positive family history is significantly associated with GERD [21,22]. This indicated that genetic inheritance played a major role in the development of GERD, as reported by many studies [23-25]. History of frequent use of analgesics, specifically NSAIDs (P = 0.003), was another risk factor (P < 0.001), which was generally comprehended due to the acidic nature of these drugs, which may boost the pathogenesis of the disorder. A questionnaire-based study of 2262 patients with GERD reported that 33% of these patients were using NSAIDs, which significantly affected GERD symptoms (P < 0.001) [26].

In terms of habitual risk factors, no significance was found in terms of being overweight or obese which was consistent with the results by Kariri et al. [8] who also reported no significance. On the other hand, Alkhathami et al. [20] in their study identified obesity as a risk factor for having GERD. Using a similar study, we found smoking, type of food, and drinks to be significant risk factors. We also found that increased frequency of having meals (>3) is a significant risk factor, unlike the results found by Alkhathami et al. [20] who stated that frequent meals were not significant. In the same context, Alrashed et al. [11] found significance in terms of eating quickly and sleeping within one hour after dinner. This indicated the importance of following healthy diets and healthy eating habits, which may significantly reduce the risk of GERD. Interestingly, it was found that exercising one to three times per week is a significant risk factor for developing GERD (OR: 2.86, CI: 1.53-5.34, P < 0.001) which was inconsistent with the results of previous studies which demonstrated that increased frequencies of physical activities generally decreased the risk [11,20]. However, a recent study revealed that frequent physical activity was a risk factor in obese patients, and not in patients with a low BMI [27]. A possible explanation for this is that frequent physical activity might cause a state of stress, which can compress gastric contents, disturb the normal motor function of the lower esophageal sphincter, and reduce blood flow to the stomach [28-30].

Limitations to our study included the use of GerdQ as the only approach for patient assessments and the relatively small sample size of the included population. Furthermore, the Hawthorne effect might be a factor in the way the patients' behaved in filling the questionnaire. Moreover, the type of smoking, whether regular cigarettes or electronic, was not explored as it is a new trend among the population, which opens more doors to explore it in the future. Lastly, the female participants were relatively low, possibly making the study not representative regarding gender.

## Conclusions

Our study specified the results of previous studies that GERD is highly prevalent in the Saudi population. We also identified many modifiable risk factors that can significantly affect the ascendance of the disease. Awareness and public health education campaigns would help in the reduction of the disease.
